# Should genetic screening be conducted for autism spectrum disorder?A case report of a 20 months old child

**DOI:** 10.1192/j.eurpsy.2025.1125

**Published:** 2025-08-26

**Authors:** E. Dogan Elmas, C. Azizagaoglu, F. O. Ciftci, S. Bodur

**Affiliations:** Child and Adolescent Pscyhiatry, Gülhane Training and Research Hospital, Ankara, Türkiye

## Abstract

**Introduction:**

Autism spectrum disorder(ASD) is a developmental disorder that includes social communication challenges, restricted interests, repetitive behaviors, and intellectual incapacity. The specific etiology of ASD is unknown. However, it is thought to be a combination of genetic and environmental factors. ASD has been linked to copy number variation (CNV), which alters chromosome structure at the submicroscopic level. 16p11.2 deletion is one of the most commonly documented cytogenetic causes of ASD, with an estimated incidence of 0.5% among ASD patients. (Ju et al. 2021; Frontiers in cellular neuroscience, 15, 718720.)

**Objectives:**

We investigated to explain the hereditary characteristics of a case of ASD that included intellectual disability and dysmorphic facial traits.

**Methods:**

Our patient is 20 months old and applied to our clinic accompanied by his parents due to speech delay and with the story of forgetting a few words he had learned around 1 year old. When we evaluate it from a developmental perspective, he held his head at the 10th month, babbled at the 8th month, sat without support 13 the month, crawled at the 15th month, walked at the 17th month, said his first words at the age of one, but he has regression history around 1 year and 4 months old, and not completed his toilet training yet. At 18 months old, a genetic screening was conducted due to brachycephalic facial structure, prominent forehead ridge and protrusion in the frontal area, mild midface hypoplasia, hypertelorism (interocular distance of 2.9 cm), slightly slanted eye sockets, synophrys, anteverted nostrils, a small nose with a deeply set nasal bridge, mild prognathism, and deeply set, posteriorly rotated ears. The genetic screening revealed a 597.84 kb microdeletion in the short arm of chromosome 16(16p11.2) through CGH array analysis

**Results:**

During our examinations, it was observed that the child is generally passive, does not sufficiently use verbal and non-verbal communication, has inadequate eye contact, and responds inconsistently to their name. Based on the developmental tests and our evaluation, the child was found to be significantly behind in developmental milestones, leading to a consideration of autism and cognitive delay. It was recommended that the child begin special education.

**Conclusions:**

The deletion of 16p11.2 may lead to developmental disorders and poor socio-cognitive performance by disrupting long-range prefrontal synchronization. This is supported by ASD-associated CNV and impaired macroscale connectivity. (Bertero et al. 2018; Brain, 141(7), 2055-2065) Deletions of 16q11.2 are associated with higher rates of psychopathology relative to familial controls, emphasizing the need for early identification, diagnosis, and intervention (Niarchou et al,2019; Translational psychiatry; 9(1), 8)

**Disclosure of Interest:**

None Declared

